# Supplementary material for: Extracting random numbers from quantum tunnelling through a single diode
Source: Sci Rep. 2017 Dec 19;7:17879. doi: 10.1038/s41598-017-18161-9 (PMC5736612; doi:10.1038/s41598-017-18161-9)
Supplement: Supplementary file 1 — Supplementary Information [file 41598_2017_18161_MOESM1_ESM.doc]

Supplementary information: Extracting random numbers from quantum tunnelling through a single diode

Ramón Bernardo-Gavito[[1]](#footnote-2), Ibrahim Ethem Bagci[[2]](#footnote-3), Jonathan Roberts^1^, James Sexton[[3]](#footnote-4), Benjamin Astbury^1^, Hamzah Shokeir^1^, Thomas McGrath^1^, Yasir J. Noori^1^, Christopher S. Woodhead^1^, Mohamed Missous^2^, Utz RoedigError: Reference source not found and Robert J. Young1,[[4]](#footnote-5)*

**Randomness extraction**

Frauchiger et al.^1^ provide a framework to remove negative effects of imperfect quantum processes on QRNGs. Practical implementations of QRNGs are always subject to noise. This noise cannot be controlled and is not guaranteed to be random. Proposed framework applies randomness extraction to the result of the device to get true randomness. Randomness extraction consists of block-wise two-universal hashing. Here the result of the device is divided into n-bit sized blocks, and each block is given to a hash function to get l-bit sized new blocks. New blocks are then concatenated to get the final result, and l is generally smaller than n. With the careful selection of the parameters n and l as well as two-universal hashing algorithm, truly random blocks can be generated. The paper includes efficient implementation of proposed randomness extraction, which we used in this work.

**NIST tests**

In order to check the viability of the proposed random number generators as a part of a cryptographic scheme we performed the battery of tests included in the NIST randomness test suite^2^. Running the tests on the raw data extracted directly from the RTD shows that the sequences of bits are locally random but present a long-term lack of randomness due to the thermal fluctuations described in the main text.

On the other hand, when the data is distilled using the double hash function method we obtain results that are compliant with the NIST randomness standards for cryptographic applications. The code used for the randomness extraction is described in the previous section. Table S1 (provided as a MS Excel spreadsheet) shows the results of the tests ran on 50x10^6^ raw bits. The output after applying the randomness extraction code consists in 16.5x10^6^ bits and is divided in 30 datasets of 550000 bits. The output of the test suite is shown in the supplementary dataset Excel file.

For the longest runs of ones test we used N=100 substrings with a length of M=10000 bits. Non-overlapping templates test was ran using a sequence length of n=1000000, block length m=9, substring length M=125000, N=8 substrings. For the overlapping template of all ones tests, these values were n=1000000, m=9, M=1032, and N=968. The linear complexity test used a substring length of M=500 and N=2000 substrings. The sequence length for the random excursion test and its variant was n=1000000, with varying number of cycles depending on the sequence. The rank tests use its default value of 97 matrices for the given data size. Serial test used a block length of m=16 for sequences of n=1000000 bits. The Mauer’s universal statistical test used the default values of L=7, Q=1280, and K=141577 for the given input. For the universal entropy test we used a block length of m=10 bits with sequences of n=1000000 bits. The significance level was set to α=0.05 (5%), as it is the most widely used value for this kind of tests.

As stated in the NIST randomness tests suite, he minimum pass rate for each statistical test except for the random excursion (variant) test is approximately is 24 for a sample size of 30 binary sequences. The minimum pass rate for the random excursion (variant) test

is approximately 10 for a sample size of 14 binary sequences. Al the rests that were run are successful considering both the proportion and the P-value criteria as described in the NIST randomness test suite manual^2^.

1. Physics Department, Lancaster University, Lancaster, LA1 4YB, UK [↑](#footnote-ref-2)
2. School of Computing and Communications, Lancaster University, Lancaster, LA1 4WA, UK. [↑](#footnote-ref-3)
3. School of Electrical and Electronic Engineering, University of Manchester, M13 9PL, UK. [↑](#footnote-ref-4)
4. ^*^ Correspondence should be addressed to R.J.Y. (email: [r.j.young@lancaster.ac.uk](mailto:r.j.young@lancaster.ac.uk)) [↑](#footnote-ref-5)
